# Q fever transmission mediated by ticks

**DOI:** 10.1080/22221751.2025.2572680

**Published:** 2025-10-08

**Authors:** Francisco Tejerina, Mercedes Marín, José A. Oteo, Pablo Martin-Rabadán, Patricia Muñoz, Leire Pérez, Teresa Aldámiz, Chiara Fanciulli, Cristina Diez, Paula Santibáñez, Aránzazu Portillo

**Affiliations:** a Clinical Microbiology and Infectious Diseases Department, Hospital General Universitario Gregorio Marañón, Madrid, Spain; b Instituto de Investigación Sanitaria Gregorio Marañón, Madrid, Spain; c CIBERINFEC, CIBER de Enfermedades Infecciosas, Instituto de Salud Carlos III, Madrid, Spain; d Centre of Rickettsioses and Arthropod-Borne Diseases (CRETAV), San Pedro University Hospital-CIBIR, Logroño, Spain; e Departamento de Medicina, Facultad de Medicina, Universidad Complutense de Madrid, Madrid, Spain; f CIBERES, CIBER Enfermedades Respiratorias, Instituto de Salud Carlos III, Madrid, Spain

**Keywords:** coxiel

## Abstract

We report a patient diagnosed with Q fever who developed an acute hepatitis after tick exposure. Sequence analysis of PCR fragments from the patient´s blood and the tick from the patient (*Hyalomma lusitanicum*) showed homologous (100% identity) *Coxiella burnetii* sequences, suggesting direct Q fever transmission by a *H. lusitanicum* tick.

*Coxiella burnetii*, phylum Proteobacteria, gamma subdivision, family Coxiellaceae, and order of *Legionellales*is, is the agent responsible for Q fever. Acute presentation of Q fever usually manifests as a flu-like syndrome, pneumonia, and/or hepatitis. Transmission is mainly airborne, but several facts suggest other sources of infection, such as ticks that could play a significant role in transmission [1]. Detection of *C. burnetii* in different tick species [2], documentation of transmission mediated by ticks in experimental animal models [3], and reports of Q fever episodes with indirect relationship with tick bites highlight that tick exposure could be a source of transmission in some cases of Q fever [4,5]. Despite all these facts, there has been no direct confirmation of *C. burnetii* transmission from ticks to humans.

We report a patient diagnosed with Q fever who developed acute hepatitis after tick exposure. Sequence analysis of PCR fragments detected in the patient´s blood and in the tick recovered from the patient (*Hyalomma lusitanicum*) showed 100% sequence identity of *C. burnetii*, suggesting direct transmission of Q fever by a *Hyalomma* tick specimen.

## The study

On 5 June 2023, a 63-year-old woman, with no significant medical records, was admitted to Hospital General Universitario Gregorio Marañón (Madrid, Spain) with a 6-day history of an acute febrile syndrome. Twelve days prior to evaluation (25 May), she had been hiking in a rural area near Madrid and after a 72-h asymptomatic period, she had noticed swelling on her lower left abdomen and had removed a tick. Four days after the tick´s removal, she started with fever of 39°C and systemic symptoms. Physical examination at admission revealed an approximately 0.5-cm erythematous macule on her lower left abdomen, where she recalled removing a tick. The patient exhibited anaemia, thrombocytopenia, and elevated hepatic cytolytic enzymes (Supplementary Table 1). She provided the arthropod that had been kept refrigerated. Morphological identification revealed it was a *Hyalomma* tick specimen. Empirical treatment with ceftriaxone and doxycycline was started.

Considering the history of tick-bite, a whole blood sample obtained the same day the tick was provided by the patient, was sent to the Microbiology National Referral Center (CNM) (Madrid, Spain) for RT–PCR screening of Crimean-Congo haemorrhagic fever virus with negative results. At admission, indirect chemiluminescence antibodies immunoassays against *Rickettsia* spp. and *Borrelia burgdorferi* sensu lato (VirClia, Vircell Microbiologists, Granada, Spain) were also negative. A multiplex real-time PCR for *Anaplasma phagocytophilum*, *B. burgdorferi* s.l.*/Borrelia miyamotoi/Borrelia hermsii,* and *C. burnetii* (Viasure, Certest Biotec, Zaragoza, Spain) was performed in EDTA-blood with positive results to *C. burnetii*. This finding was confirmed with a specific *C. burnetii* homemade real-time PCR targeting the IS1111 region, which is specific for *Coxiella burnetii*, using a TaqMan probe.

This PCR assay is routinely used in our department to diagnose *C. burnetii* infective endocarditis directly from heart valve tissue. It has been validated using a collection of heart valves that tested positive for *C. burnetii* by *16S rRNA* PCR and sequencing. Each PCR run includes positive and negative controls, as well as amplification of a human *β-globin* gene fragment as an internal control. The specificity of each positive *IS1111* PCR result is confirmed by Sanger sequencing. A serum sample sent to CNM for indirect immunofluorescence assays showed titers of IgG anti-phase I: 1/200, IgG anti-phase II: 1/800; IgM anti-phase I: not detected, IgM anti-phase II: 1/50; IgA anti-phase I: not detected, and IgA anti-phase II: not detected.

The patient received treatment with doxycycline for 14 days presenting fever disappearance and ALT/AST normalization.

The case was discussed with members of the Centre of Rickettsioses and Arthropod-Borne Diseases (CRETAV) at the San Pedro University Hospital-CIBIR, Logroño (Spain), to investigate the possible connection of Q fever and the tick-bite with at least 48 h of persistence in the patient. Remaining extracted DNA and an aliquot of the blood sample, as well as the tick, were sent to CRETAV for arthropod identification, PCR investigation of *C. burnetii* in the tick and evaluation of the potential genetic relationship of *C. burnetii* sequences from the tick and the patient’s blood sample.

At CRETAV, morphological analysis of the arthropod identified a fragmented female of *Hyalomma lusitanicum*, and its genetic analysis confirmed the species identification. The 16S ribosomal RNA sequence of the tick showed the highest similarity (99.7%; 403/404 nt) with that of *H. lusitanicum* cell line HLE/LULS48 (GenBank accession no: ON366981). In addition, *C. burnetii* was detected in the tick and in the patient’s blood by qPCR of *IS*30a. Conventional PCR assays targeting *IS*1111, *htp*-AB, *rpoB,* and *groEL* genes also yielded positive results. Nucleotide sequence analysis of the obtained amplicons from the tick and the blood confirmed the specific detection of *C. burnetii* and revealed that they shared significant similarity (≥99.5%) suggesting they are the same strain (GenBank accession number: CP018150) (Table 1).

**Table 1.** Comparison of Coxiella burnetii sequences obtained from tick and patient.

PCR target gene (type of PCR)	% nucleotide identity (nt) with *Coxiella burnetii*	
	In the tick	In patient’s samples
*IS*30a (qPCR)	100% (122/122 nt)	100% (104/104 nt) [DNA]
*IS*1111 (cPCR)	99.8 (642/643 nt)	100% (643/643 nt) [whole blood-EDTA and DNA]
*groEL* (nested cPCR)	100% (610/610 nt)	100% (575/575 nt) [whole bood-EDTA]
*htp*-AB (cPCR)	99.5% (217/218 nt)	Inconclusive sequence^a^
*rpoB* (cPCR)	100% (564/564 nt)	Inconclusive sequence^a^

^a^ PCR products were not concentrated enough for direct sequencing via cPCR.

qPCR: real-time PCR; cPCR: conventional PCR; nt: nucleotide.

## Discussion

The possibility of *C. burnetii* transmission by ticks and Q fever has been suggested, although no human cases of direct Q fever transmission from infected ticks have been demonstrated. It is known that ticks may shed high concentrations of *C. burnetii* through faeces and to a lesser extent, through saliva and/or coxal fluids [1, 6]; anyway, *C. burnetii* is a highly virulent human pathogen, and a few bacteria may cause disease [7].

A relevant number of tick species are competent vectors for *C. burnetii* transmission under experimental assays, including *Hyalomma* species, such as *H. aegyptium* [8]. Experimental studies of the transmission of *C. burnetii* to vertebrates are lacking for *H. lusitanicum*. This three-host species predominates in warm, open, and dry habitats, and is the most abundant exophilic tick in central Spain with expanding populations across the country. Immature tick stages parasitize wild rabbits and hares while adults, with peak activity in spring and summer and aggressive behaviour, primarily infect red deer and wild boar. This cycle occurs in parallel to the classic tick domestic cycle involving cattle, sheep, and goats, hosts that mainly contribute to environmental contamination and aerosolization of infectious particles of *C. burnetii*. In addition, transovaric and trans-stadial transmission of *C. burnetii* and the ability to establish natural transmission cycles with wild mammals have been reported for this species in Spain. *H. lusitanicum* acts as a bridge between wildlife, livestock, and humans. These bioecological characteristics support the potential role of *H. lusitanicum* in the natural transmission of *C. burnetii* in Iberian ecosystems [9,10].

According to different studies, the prevalence of *C. burnetii* in ticks may vary, but these results may be overestimated due to the presence of *Coxiella-*like endosymbionts in ticks [8]. These bacteria, with no demonstrated pathogenic capacity, can be misidentified as *C. burnetii* by PCR assays without subsequent sequencing [8]. Herein, all amplicons were sequenced in both senses and the sequences confirmed the presence of *C. burnetii*-specific DNA in the patient’s blood and in the tick (Table 1). Moreover, for *H. lusitanicum*, there are no descriptions of associated *Coxiella*-like endosymbionts; instead, *Francisella*-like bacteria seem to be the primary symbionts of this tick species [11].

Although different ticks seem able to transmit Q fever, they may inefficiently transmit to humans, and there are only scarce reports of Q fever transmission to humans mediated by ticks [4,5,12-15]. To the best of our knowledge, in all these cases tick transmission could not be directly confirmed, and infection was presumed based on a previous exposure to ticks or a concomitant infection with another pathogen transmitted by ticks, but in all these cases, airborne or other modes of transmission could not be excluded.

Here we report a patient exposed to a tick-bite, for a period sufficient to transmit other pathogens transmitted by tick exposure, who developed Q fever without any other concomitant infection mediated by tick transmission. The patient also recovered the tick, which allowed us to evaluate the presence of *C. burnetii* in this sample. Nucleotide sequencing of PCR targets obtained from the patient’s blood and the *Hyalomma* tick confirmed they shared the same nucleotide sequences. This fact suggests acquisition of the bacteria through the tick-bite, since the patient manipulated the tick to remove it, exposure to *C. burnetii* by inhalation of the infected tick feces or intradermal inoculation of the agent by tick contact cannot be totally ruled out.

## Supplementary Material

Suppl Table 1 tick transmission.docx
