# Supplementary material for: Q fever transmission mediated by ticks
Source: Emerg Microbes Infect. 2025 Oct 8;14(1):2572680. doi: 10.1080/22221751.2025.2572680 (PMC12536617; doi:10.1080/22221751.2025.2572680)
Supplement: Suppl Table 1 tick transmission.docx [file TEMI_A_2572680_SM3918.docx]

**Suppl Table 1. Blood analysis on admission and follow up**

|  | **Reference range** | **05/06/2023** | **08/06/2023** | **12/06/2023** | **28/06/2023** | **29/09/2023** |
| --- | --- | --- | --- | --- | --- | --- |
| **Hemoglobin (g/dL)** | 12-16 | 11.50 | 10.70 | 10.80 | 11.50 | 12.60 |
| **Platelet count (×10^3^/uL)** | 140-400 | 83 | 149 | 377 | 190 | 222 |
| **White-cell count (×10^3^/uL)** | 4-10 | 3.35 | 4.27 | 7.32 | 5 | 5.50 |
| **ALT (U/L)** | 5-31 | 217 | 792 | 641 | 167 | 50 |
| **AST (U/L)** | 10-31 | 198 | 680 | 279 | 94 |  |
| **Bilirubin (mg/dL)** | (0.1-1.1) | 1.22 | 0.42 | 0.50 | 0.69 | 0.56 |
| **GGT (U/L)** | 6-40 | 55 | 62 | 72 | 43 | 20 |
| **LDH (U/L)** | 135-214 | 746 | 862 | 447 | 304 | 240 |
| **PCR (mg/L)** | 0-5 | 139.20 | 92.50 | 15.10 | 4.70 |  |
